# Computer simulations of a heterogeneous membrane with enhanced sampling techniques

**DOI:** 10.1063/5.0014176

**Published:** 2020-10-13

**Authors:** Yevhen K. Cherniavskyi, Arman Fathizadeh, Ron Elber, D. Peter Tieleman

**Affiliations:** 1 Department of Biological Sciences and Centre for Molecular Simulation, University of Calgary, 2500 University Drive NW, Calgary, Alberta T2N 1N4, Canada; 2 The Oden Institute for Computational Engineering and Sciences, University of Texas at Austin, Austin, Texas 78712, USA; 3 Department of Chemistry, University of Texas at Austin, Austin, Texas 78712, USA

## Abstract

Computational determination of the equilibrium state of heterogeneous phospholipid membranes is a significant challenge. We wish to explore the rich phase diagram of these multi-component systems. However, the diffusion and mixing times in membranes are long compared to typical time scales of computer simulations. Here, we evaluate the combination of the enhanced sampling techniques molecular dynamics with alchemical steps and Monte Carlo with molecular dynamics with a coarse-grained model of membranes (Martini) to reduce the number of steps and force evaluations that are needed to reach equilibrium. We illustrate a significant gain compared to straightforward molecular dynamics of the Martini model by factors between 3 and 10. The combination is a useful tool to enhance the study of phase separation and the formation of domains in biological membranes.

## I. INTRODUCTION

Biological membranes are complex environments that function as a semi-permeable barrier between the cell interior and the external environment. They consist of phospholipids, cholesterol and protein molecules, and more. The membrane components assemble into microphases and nanodomains and regulate cell function. According to the raft hypothesis, lateral inhomogeneity of the lipid membranes plays a key role in cell signaling, protein aggregation, and membrane fusion.^1^

A number of experimental techniques such as x-ray and neutron scattering,^2,3^ nuclear magnetic resonance (NMR),^4^ and others^5^ provide structural data on lipid membranes. Despite significant progress, studying the structure of biological membranes at molecular resolution remains a challenging task due to the disordered and fluid characteristics of these systems. Scattering-based techniques can directly probe the spatial organization of lipid membranes without introducing additional probes that alter the membrane structure.^6^ However, the scattering signal is a spatial and temporal average over many fluid structures, leading to a smooth and less detailed signal. The averaging and separation of signals is even more difficult in mixed membranes with multiple types of phospholipids.^7^

The rapid growth in the power of computers and the development of simulation methodology has significantly increased the use of computer simulations for the study of lipid membranes.^8–10^ Recently, a state-of-the-art realistic model of the plasma membrane that contains more than 60 types of phospholipids was developed.^11^ Nevertheless, sampling the equilibrium distribution of constituents of heterogeneous membranes remains computationally challenging. The key problem is the slow diffusion of phospholipids in the membrane plane that prevents efficient mixing at time scales accessible for Molecular Dynamics (MD). One approach to reducing the computational cost is to use a coarse-grained description of lipids, such as the Martini model.^12^ On average, the Martini model represents four heavy atoms as a single particle or a bead. The reduction in the total number of particles compared to atomistic force fields leads to a significant gain in speed. Moreover, the removal of the fast degrees of freedom (e.g., vibrations of atomic bonds) enables the use of larger time steps and diminishes thermal noise and friction. The diffusion coefficient is D = k_B_T/γ, where k_B_ is the Boltzmann constant, T is the temperature, and γ the friction coefficient. Hence, the diffusion is faster when the friction coefficient is smaller. The effective speedup of the diffusion within the Martini model (∼4–5 times faster than in atomistic models and experiments) was documented in the literature.^12–14^ However, even with the significant speedup compared to atomistic models, reaching equilibrium of large heterogeneous membranes with classic MD and the Martini force field is computationally expensive.

Recently, a sampling approach that combines MD with Monte Carlo (MC) approaches in the grand canonical ensemble was proposed. The method is particularly suitable for the simulation of heterogeneous lipid membranes in which the different lipids are quite similar.^15^ The system is sampled by alternating steps of (1) a straightforward MD step in the microcanonical or canonical ensemble and (2) an MC move that replaces a phospholipid by another phospholipid of a different type. A random lipid is selected for such an alchemical transformation. If the MC move is accepted following the usual Metropolis criterion, the lipid molecule is modified to its lipid counterpart (for example, from DPPC to DPPS). The MD/MC approach is not bound by the slow lateral diffusion of lipids, and phase separation and mixing are potentially sampled more efficiently than straightforward MD. The challenge with the MC approach is that the lipid types must be similar for the MC move to be accepted with a reasonable probability. Because the acceptance is typically low in Monte Carlo with Molecular Dynamics (MC-MD) in membranes, many trials are needed. To increase the number of trials for a fixed number of force evaluations only a single or a few MD steps separate two MC steps. The extraction of kinetic information (such as the diffusion constant) is not possible in this approach. The rapid transitions between MD and MC moves may also lead to hysteresis, and the sampling may deviate from the desired equilibrium. In addition, as the main goal of this approach is to enhance the sampling of mixing lipids, the procedure has to be more efficient than straightforward MD to be of any practical use.

Optimizing trial MC moves to achieve higher acceptance probabilities allows longer MD trajectories between the MC moves and better relaxation to equilibrium. It also makes it possible to extract short time kinetic information, such as local diffusion constant. Therefore, a new algorithm was proposed: Molecular Dynamics with Alchemical Steps (MDAS).^16^ Instead of performing the exchange in a single MC move, we conduct a gradual growth of the two lipids into their counterparts, relying on the Jarzynski equality,^17^ and the algorithm for candidate Monte Carlo moves^18^ to obtain the correct statistics. This exact approach significantly increases the acceptance probability of the MC move. Instead of modifying a single phospholipid, an exchange of a pair of phospholipids of different types is considered, which ensures a fixed composition.

**FIG. 1.**
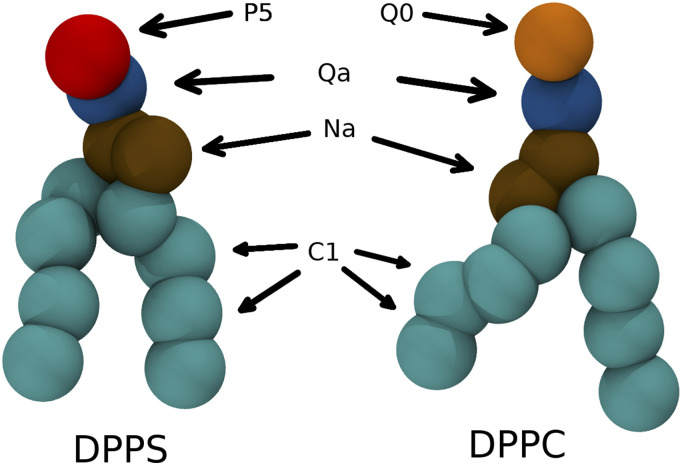
DPPC and DPPS lipid molecules in the Martini model. On average four heavy atoms with corresponding hydrogens are represented as one bead. Different bead types are marked on the figure and highlighted with different colors. The only difference between DPPC and DPPS lipid molecules in the Martini force field is the type of one headgroup bead.

The MDAS algorithm generates steps that are more likely to be accepted than conventional MC moves. However, if the interactions of the exchanged phospholipids with their environments are significantly different, the gradual modification of the molecules can be inefficient. An example of a challenge for atomically detailed simulation, which is discussed in Sec. IV, is the pair of PS and PC phospholipids. PC is neutral, while PS is negatively charged. Therefore, the electrostatic interactions impact the rate of relaxation to equilibrium, leading to many rejected MC-MD and MDAS steps. This makes the efficient use of this procedure a challenge for atomistic simulations. However, this problem may not be present in the coarse-grained model of Martini. The mutation of PS to PC in Martini adjusts one bead (the head group) with only short-range interactions (Fig. 1).

Here, we explore the use of the MDAS and MC-MD algorithms specifically for the sampling of a lipid mixture with the Martini force field. We test the performance of the algorithm with a binary DPPC/DPPS mixture and benchmark it against straightforward MD and MC-MD approaches. For comparison, we also carry out the atomistic equivalent.

## II. THEORY

### A. MDAS and MC-MD

In the current paper, we use three approaches for the simulation of the system—straightforward MD, MC-MD, and MDAS. The MC-MD simulation is conducted as a series of alternating straightforward MD steps and MC lipid exchange moves. We randomly select a pair of different types of phospholipid molecules (e.g., one DPPC and one DPPS molecule) and change the chemical identity of the two lipids. Then, we either accept or reject the proposed MC move based on the Metropolis criterion,

$$\begin{array}{ccc} \begin{array}{c} P_{accept}=\text{min}\left[1,\text{exp}\left(\frac{-\Delta U}{kT}\right)\right],\\ \Delta U=U\left(r_{1},\ldots ,r_{i},\ldots ,r_{j},\ldots \right)-U\left(r_{1},\ldots ,r_{j},\ldots ,r_{i},\ldots \right), \end{array} & & \end{array}$$ (1)

where ΔU is the energy difference before and after the trial move, $r_{i}$ and $r_{j}$ are the coordinates of the selected pair of lipids, respectively, k is the Boltzmann constant, and T is the temperature. If the move is accepted, we continue with an MD trajectory starting from the new state. If the move is rejected, we go back to the state before the exchange attempt and run a straightforward MD step.

The MDAS algorithm substitutes a single-step MC move with a gradual adjustment, which is computed using an alchemical trajectory (AT).^16^ During the AT, the selected phospholipid molecules change into their counterparts (e.g., DPPC to DPPS and vice versa). MDAS simulation consists of (1) straightforward MD trajectories for sampling and (2) “alchemical” trajectories (AT) that modify the phospholipids and generate candidate MC moves.^18,19^ The AT is similar to the alchemical methods used to determine the free energy difference between two states.^20,21^ Like in a free energy calculation, the AT path is parameterized with λ ∈ [0, 1]. When λ = 0, we are at the beginning of the attempted exchange, and when λ = 1, at the end of it. The potential energy along the AT, *U*(*R*, *λ*), is a function (*λ*). The dependence of the potential on λ is the choice of the user. The simplest implementation is linear. For an exchange of a system A to a system B, we have *U*(*λ*) = (1 − *λ*)*U*_*A*_ + *λU*_*B*_. We conduct the AT as follows: We run M steps of straightforward MD at a fixed value of λ and then increase λ by a small Δλ in a single step. Starting with λ = 0, the M steps and the increase in λ are repeated until λ is equal to 1. The work done on the system during the entire AT is

$$w\left(\lambda =0\to \lambda =1\right)=\sum_{i}\left(U_{\lambda_{i}+\Delta \lambda }\left(x_{i}\right)-U_{\lambda_{i}}\left(x_{i}\right)\right),$$ (2)

where *x*_*i*_ are the system coordinates after *i* repeats of the M steps. The total work is used in an acceptance–rejection criterion of the AT move^16,19^ similar to Metropolis [Eq. (1)],

$$P_{accept}=\text{min}\left[1,\text{exp}\left(\frac{-w}{kT}\right)\right].$$ (3)

If the move is accepted, we continue the simulation from the last configuration of the state λ = 1 (the lipids are exchanged) and proceed with another segment of a straightforward MD trajectory. If the move is rejected, we discard the AT and continue from the last step of the previous straightforward MD segment. Thus, the entire MDAS simulation consists of a series of short conventional MD trajectories and exchange steps (AT) in between. We use only the conventional MD segments to calculate the thermodynamic properties of the system.

**FIG. 2.**
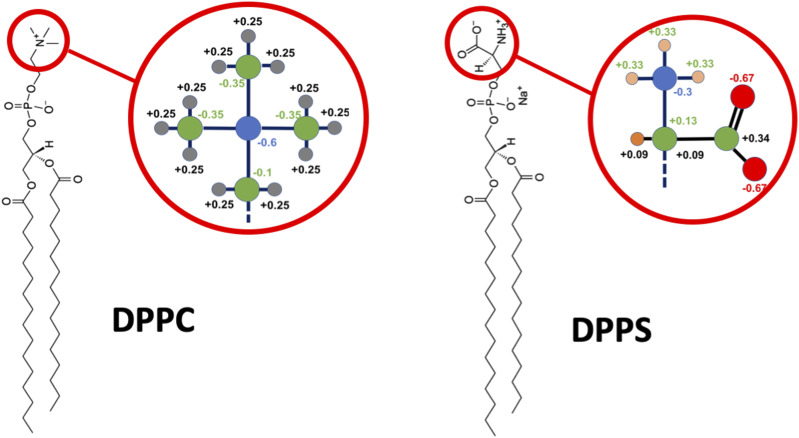
Structures of DPPC and DPPS lipid molecules with required alchemical changes in the atomically detailed MDAS simulations.

In the current paper, we use two force fields to study the DPPC/DPPS lipid mixture—coarse-grained Martini force field and atomically detailed CHARMM36 force field. In the simulations with the Martini model, an exchange move consists of changing the chemical identity of a single headgroup particle (bead) that corresponds to the transition of the type P5 bead of DPPC to type Q0 bead of DPPS (Fig. 1). The change is performed by the modification of the VdW interaction parameters and charge without any particles vanishing or new particles appearing. With the atomistic CHARMM36 force field, an exchange move requires a swap between the choline group of DPPC and the serine group of DPPS, which involves multiple atoms (Fig. 2). In contrast to the Martini case, this transition involves appearance/disappearance of atoms. In both models, the remaining parts of the lipid molecules are left unperturbed during an exchange move.

The specific MDAS choice of Δλ = 1 and M = 0 is equivalent to a single-step MC exchange. In MDAS, the role of the AT trajectory is to allow the system to relax. A gradual exchange with Δλ ∼ 0.001–0.01 yields significantly lower work compared to the direct MC exchange, as it produces less steric overlap and corresponding high energies. Therefore, the MDAS steps in the atomically detailed models are accepted with a much higher probability than direct MC. However, if the two types of phospholipids are similar (as DPPC and DPPS are in Martini), the additional cost of computing AT vs direct MC is not *a priori* an improvement and testing is required.

## III. METHODS

The simulations were performed with the standard Martini 2.2 force field,^22^ with and without polarizable water.^23^ The electrostatic interactions were modeled with the reaction-field method.^24^ The screening constant was 15 and 2.5 for non-polarizable and polarizable water, respectively. The cutoff distance of the vdW interactions was at 1.1 nm with a potential-shift modifier. All the simulations were conducted at 335 K. The temperature was fixed with velocity rescaling^25^ with 1.0 ps coupling constant and two separate coupling groups for the membrane and the solvent. A semi-isotropic (xy and z directions) Parrinello–Rahman barostat^26^ maintains a constant pressure of 1.0 bar with the coupling constant of 12.0 ps. Integration was performed with the Verlet algorithm with a 20 fs time step. Standard MD simulations were performed with GROMACS 2019.1.^27,28^

MDAS and MC-MD simulations were conducted with GROMACS 2019.1. All of the required free energy code is already available in GROMACS and the only change in the code was a hard-coded optimization to maintain lambda at the same value for *n* steps instead of the default options of a constant lambda or a lambda that changes linearly every step. For this proof of concept study, GROMACS was called a new process for every MD part and a number of scripts were used to parse the necessary energy and other information. For production use, a more integrated code is developed. A 1:1 mixture of DPPC and DPPS (200 DPPC and 200 DPPS molecules, 100 molecules of each type per monolayer) was considered. The system was solvated with 5815 Martini water beads and 265 Na^+^ and 65 Cl^−^ ions were added to the system. Because the differences in Martini between DPPC and DPPS are small (Fig. 1), a short AT of 1000 steps was sufficient. The parameter λ is modified every ten steps, hence, Δλ = 0.01. After 1000 steps, the total work is computed and the proposed move is accepted or rejected. Then, we sample another 2000 steps of straightforward MD before attempting another AT. The same approach was used for the MC-MD sampling scheme, but the AT is a single step (Δλ = 1.0). To compare different methods on equal footing, we consider the number of force evaluations used per number of sampled configurations. The cost of a single attempt of MDAS exchange is 3000 force evaluations (2000 straightforward MD and 1000 AT steps), and it is 2001 force evaluations for a single exchange attempt in MC-MD.

For comparison, we also run an atomistic MDAS simulation for the DPPS/DPPC system using the program NAMD^29^ and the CHARMM36 force field.^30^ We had considerable success in the past in simulating a mixture of DOPC and DPPC,^16^ which only differ in their tails. We show in Fig. 2 the required alchemical changes in the atomically detailed MDAS simulations. There are 26 atoms that require modifications, which is a considerably more complex task than the single-particle exchange of Martini or our previous DOPC/DPPC test case. As noted earlier, changes in the electrostatic interactions within the atomic models pose an additional and significant challenge to MDAS. The assigned charges of the phospholipids are of the CHARMM 36 force field^31^ that was used in the atomistic simulations. We consider a 1:1 binary mixture of DPPC and DPPS. The bilayer consists of 200 phospholipids and is solvated with TIP3P water molecules.^32^ 100 potassium ions were added to neutralize the system. The entire system contains ∼50 000 atoms. The membrane was first equilibrated in the NPT ensemble for 10 ns, which was followed by 10 ns NVT simulation.

To examine the efficiency of MDAS, we conducted 100 AT attempts. Each AT was for a total length of 100 ps with Δ*λ* = 0.001. During an AT, the system was simulated in the NVT ensemble with a Langevin thermostat. To avoid the so-called end-point catastrophe, we used a soft-core potential with the following form to treat the van der Waals interactions during the exchange:^33^

$$U_{soft}\left(r_{ij}\right)=4\varepsilon \lambda \left[{\left(\frac{\sigma_{ij}^{2}}{r_{ij}^{2}+\delta \left(1-\lambda \right)}\right)}^{6}-{\left(\frac{\sigma_{ij}^{2}}{r_{ij}^{2}+\delta \left(1-\lambda \right)}\right)}^{3}\right],$$ (4)

where δ = 5.0 nm^2^, and λ changes from 0 to 1 during the alchemical step. Note that at λ = 1, the above expression turns into the 6-12 Lennard-Jones potential and the interaction vanishes at λ = 0. The time step was 1 fs in all the atomistic simulations. In the all-atom case, we used a dual topology scheme. We select a DPPS and a DPPC randomly and replace them with a dummy molecule that has a combination of PS/PC headgroups. Then, these molecules are being evolved with MDAS to make the exchange between the molecules. The velocities of the dummy atoms are selected randomly from the Maxwell distribution according to the desired temperature of the system. The bonded interactions of the dummy atoms do not change the statistics of the system and do not contribute to the work. If the move gets accepted, the dummy atoms and bonds are removed and a regular MD simulation is conducted before trying the next MDAS move.

## IV. RESULTS

**FIG. 3.**
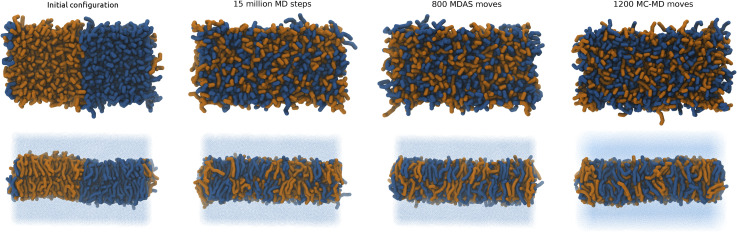
Top view and side view of the DPPC/DPPS bilayer used as a test system. DPPC lipid molecules are shown in dark blue and DPPS lipid molecules in orange. Water is represented as blue dots.

We use the 1:1 DPPC/DPPS lipid mixture as our test system for sampling efficiency. We simulate the Martini model using MDAS, mixed MD-MC, and straightforward MD. The atomically detailed calculations were attempted with MDAS. The initial state of the system was of separated DPPC and DPPS molecules (Fig. 3), which is far from the equilibrium of a uniformly mixed membrane. The radial distribution function g(r) of the PO4 beads of DPPS (or DPPC) phospholipids monitors mixing as a function of time. We have shown in Ref. 16 that the highest peak of the pair correlation function max[g(R)] is a good measure of the relaxation and is comparable to the alternative measure of mixing entropy.^34^ As the mixing occurs, max[g(r)] approaches a constant value of the uniform mixing of the two phospholipid types. To obtain a quantitative estimate of the relaxation rate, the evolution of max[g(r)] is fitted with an exponential function.

**FIG. 4.**
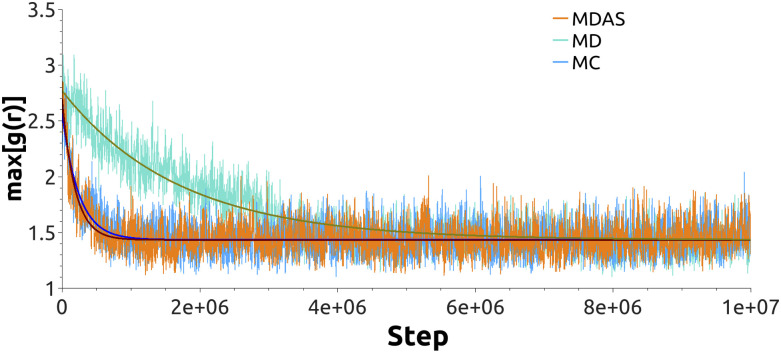
The evolution of max[g(r)] for the DPPS PO4 beads for the system with non-polarizable Martini water. Orange line—system sampled with the MDAS algorithm; turquoise—straightforward MD; light blue—MC-MD approach. Solid dark green (MD), wine (MDAS), and blue (MC-MD) lines represent exponential fits.

**FIG. 5.**
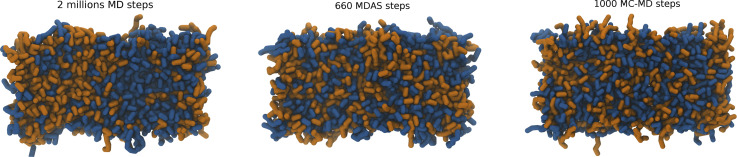
Top view of the DPPC/DPPS bilayer after 2 × 10^6^ MD steps, 660 attempted MDAS moves, and 1000 MC-MD moves. DPPC lipid molecules are shown in dark blue and DPPS lipid molecules in orange. Water molecules are not shown for clarity.

With the current choice of parameters for MDAS moves, the acceptance probability is about 29%. Figure 4 shows the time evolution of max[g(r)] for DPPS–DPPS PO4 beads as a function of the number of force evaluations. The fit of an exponential function to the evolution of max[g(r)] gives ∼11.3 ± 0.4 times speedup for the system mixing compared to straightforward MD. If we use the mixed MD-MC sampling scheme, which is equivalent to MDAS with an AT of a single step, the acceptance probability is about 16%. An exponential fit of max[g(r)] as a function of the number of force evaluations suggests ∼10.1 ± 0.2 speedup of the mixing dynamics compared to sampling by straightforward MD. Figure 5 shows snapshots of the top view of the lipid bilayer simulated with MD, MDAS, and MC-MD. After 2 × 10^6^ force evaluations in a straightforward MD simulation, the bilayer is far from laterally homogeneous. At the same time, after 660 MDAS steps or 1000 MC-MD steps, which correspond to 2 × 10^6^ force evaluations, the two lipid types are well mixed.

**FIG. 6.**
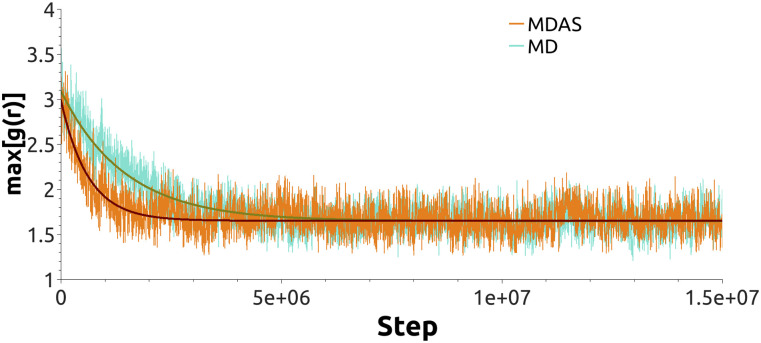
The evolution of max[g(r)] for the DPPS PO4 beads for the system with polarizable Martini water. Orange line—system sampled with the MDAS algorithm; turquoise—straightforward MD. Solid dark green (MD) and wine (MDAS) lines represent exponential fits.

To further explore the performance of the different sampling schemes with different parameterization, we simulated the same Martini system with polarizable water. As in the previous case, we benchmark MDAS and MD-MC methods against straightforward MD. With MDAS, we obtain 9% acceptance probability for the DPPC/DPPS exchange move, which translates into ∼2.5 ± 0.1 times speedup compared to straightforward MD (Fig. 6). After 10 000 attempts, we did not accept a single exchange move with MD-MC sampling. From the work values we estimate the average acceptance probability as 8.8 × 10^−5^.

**FIG. 7.**
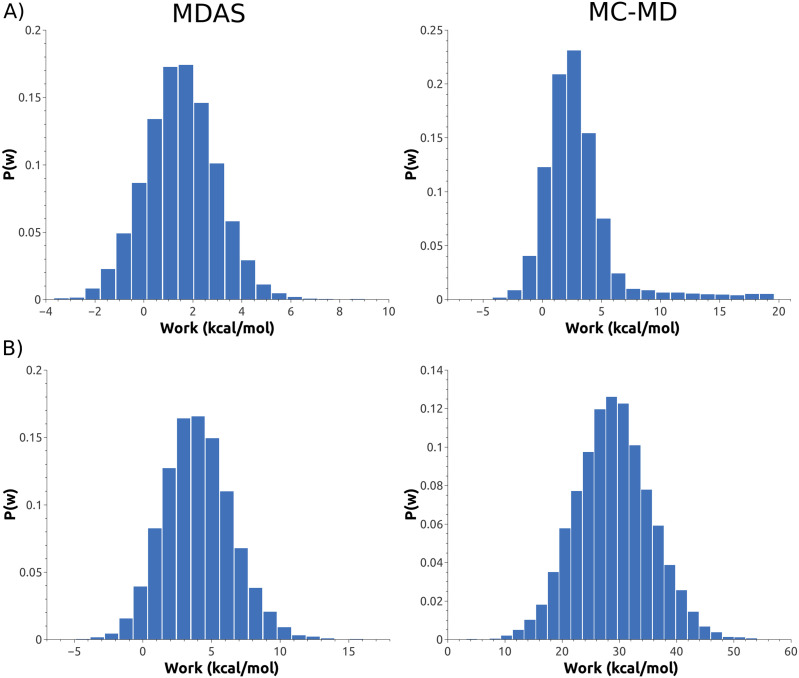
Work distribution for different types of exchange moves: MDAS (left panels) and MC-MD (right panels). Top panels (a) are the non-polarizable water in the Martini model and bottom panels (b) polarizable water.

The distribution of work for different setups with the Martini force field considered here are shown in Fig. 7. A break-down of the contributions of the VdW and Coulomb interactions to the total work for a typical MC-MD or MDAS exchange step indicates that for MDAS without polarizable water, ∼67% (0.43 kcal/mol) of total work comes from Coulomb interactions and 33% (0.20 kcal/mol) from VdW. MDAS with polarizable water gives 90% (3.49 kcal/mol) of work from Coulomb interactions and 10% (0.36 kcal/mol) from VdW. With MC-MD, we see a similar trend toward a significant increase in the weight of Coulomb interactions in the total work during an exchange move: 16% (1.07 kcal/mol) of work comes from Coulomb interactions and 84% (5.48 kcal/mol) from VdW with the non-polarizable water model. With polarizable water, MC-MD work shows 95% (26.91 kcal/mol) contribution from Coulomb interactions and 5% (1.38 kcal/mol) from VdW.

**FIG. 8.**
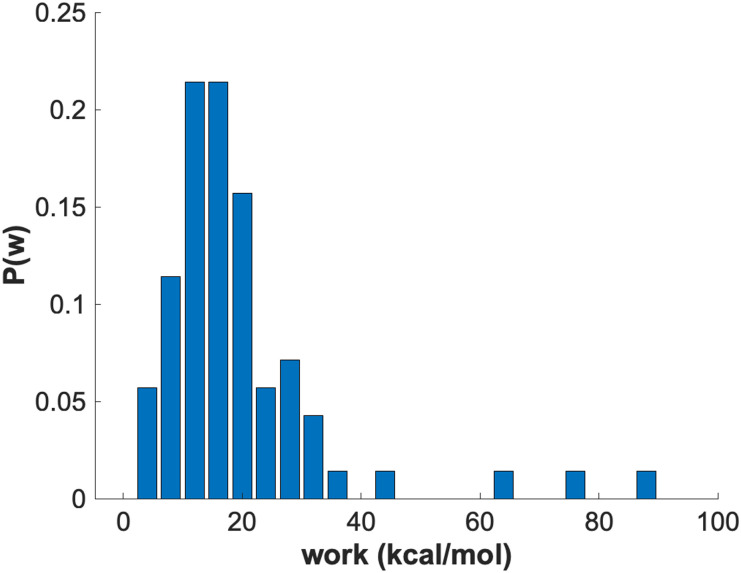
Histogram of the work values evaluated from alchemical trajectories with an atomistic force field.

For comparison, we also attempted to simulate the mixing of the DPPC/DPPS system using an atomically detailed MDAS model. We evaluated 100 AT steps of length of 100 ps (each) using *Δλ* = 0.001. The length of the straightforward MD trajectories between AT attempts was 100 ps as well. In Fig. 8, we show a histogram of the work values obtained from the AT trajectories. The distribution is broad and includes high work values, which makes the acceptance probability less than 10^−5^ and impractical for the current AT path. For a typical exchange move, the Coulomb interactions contribute ∼79% (16.2 kcal/mol) of the total work and VdW contribution is 21% (4.3 kcal/mol).

**FIG. 9.**
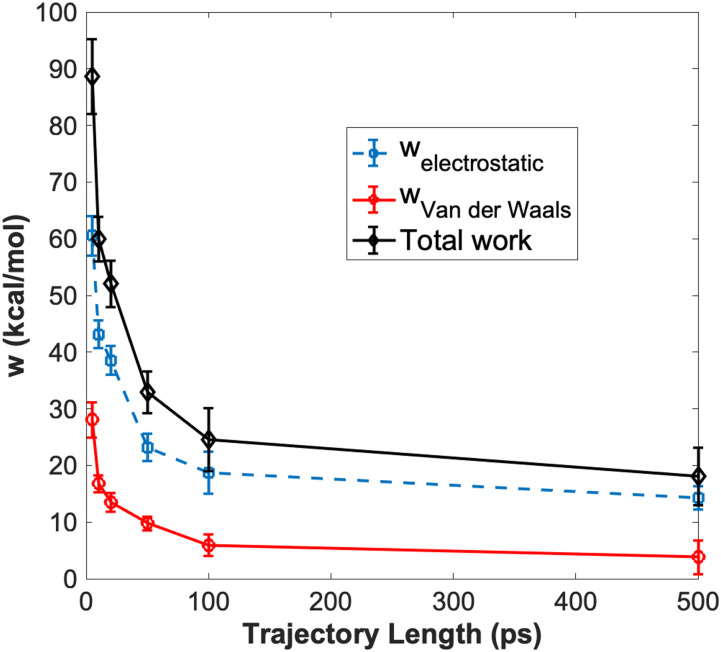
Time evolution of work as a function of the AT length for the atomically detailed model.

**FIG. 10.**
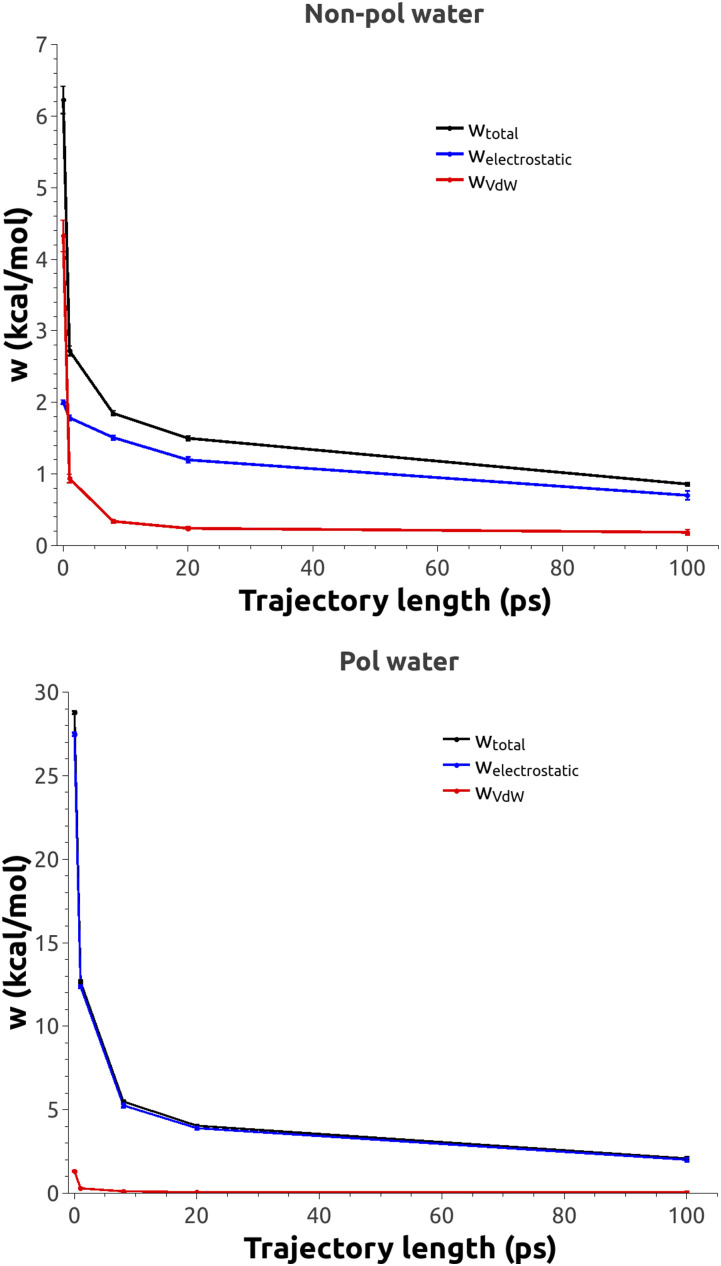
Time evolution of work as a function of the AT length for the Martini model with normal (top) and polarizable (bottom) water models.

For qualitative analysis, we have calculated time courses for the relaxation of different energy terms in MDAS and in the atomically detailed simulations (Figs. 9 and 10). For the atomistic model, the electrostatic interactions are much slower to relax than the van der Waals interactions (Fig. 9). For the Martini examples, we observe the same trend—the electrostatic interactions decay much slower than the van der Waals interactions with the AT trajectory length for both non-polarizable (Fig. 10, top panel) and polarizable water models (Fig. 10, bottom panel).

## V. DISCUSSION

The Martini model offers an efficient approach to sample membrane configurations by reducing the number of particles and using smoother energy landscapes compared to the atomistic models. It enables the study of heterogeneous membranes, assembly, and separation. However, the enormous diversity of biological membranes and their sheer sizes pose a significant challenge for converging straightforward MD simulations, even with the Martini model. MDAS enables a speedup of ∼1000 for specific atomistic systems.^16^ However, there are lipid compositions that are difficult to simulate with atomically detailed MDAS models. The challenge in MDAS simulations is the design of the AT such that the amount of work is minimal and lead to significant acceptance probability. For example, an efficient acceptance probability when using ∼100 ps trajectories of straightforward Molecular Dynamics is about 10%. This design is difficult for the exchange of phospholipids with different charges, as we illustrated in this manuscript for the DPPC/DPPS system. The charge and the membrane electric field in atomically detailed models relax slowly to the new equilibrium imposed by the exchange. Here, we have shown that the combination of Martini and MDAS is promising. The simplified description of the electrostatic interactions and the smaller differences between two different lipid topologies facilitate the design of exchange pathways and a high acceptance rate of transformation steps (Figs. 7 and 8). The design of an efficient AT for diverse pairs of phospholipids is a topic of ongoing research.

We compared straightforward MD and MDAS calculations for an atomistic model and the standard and polarizable Martini models. MDAS has the potential to be significantly advantageous compared to straightforward MD (Figs. 4 and 6), in particular for the standard Martini model. The efficiency of the MDAS algorithm can be evaluated by the computed work distributions from exploratory ATs (Figs. 7 and 8). An acceptance probability of the order of or greater than 10% allows for straightforward MD trajectories of about 100 ps long between ATs. If the acceptance probability is below this threshold, straightforward MD is likely more efficient.

What types of AT generate small values of work? In our experience, modifications of the hydrocarbon chain (lengths, or single and double bond along the lipid chain) are good candidates for an MDAS calculation in atomic detail. A modification of the head group is more challenging for atomically detailed models, both compared to standard MD and compared to Martini (Figs. 7 and 8). For phosphate head groups of different charges, an efficient AT is hard to find. Similarly, an inefficient MC-MD move is found in the Martini model that incorporates electrostatics of water [Fig. 7(b), right panel].

The DPPC/DPPS Martini system with polarizable water is an interesting example in which the MC-MD algorithm (or a single step AT) is not very efficient. In the non-polarizable case, the charged PS headgroups interact electrostatically only with the ions as the water beads in the Martini model are not charged. However, more electrostatic interactions are present when the polarizable water model is used. The water model includes an induced dipole that interacts with the charges of the head group. When we switch from PC to PS in a single step, the electrostatic interactions of the PS groups with water molecules contribute to a significant energy difference between the exchanged states, which amounts to 95% (26.9 kcal/mol) of the total work done during an exchange (see the breakdown of the different contributions to the total work in Sec. IV). As a result, the average acceptance probability in MC-MD is ∼8.8 × 10^−5^. However, if we simulate the transition between the PS/PC headgroup gradually with an AT, the work of the transition is reduced [Fig. 7(b), left panel], which translates into a higher acceptance probability of the proposed exchange move. The electrostatic interaction still contributes up to 90% of the total work in this case, but the contribution amounts to ∼3.5 kcal/mol in contrast to 26.9 kcal/mol with MC-MD for a typical exchange move. The VdW contribution to the total work is increased in the absolute value in the case of polarizable water for MDAS (from 0.20 kcal/mol to 0.36 kcal/mol) and decreased for MC-MD (from 5.47 kcal/mol to 1.37 kcal/mol), but the total work is still dominated by the electrostatic interactions. As the only source of new charges in the setup with polarizable water, compared to the non-polarizable case, is the dipoles of the water beads, we attribute the difference between work with MDAS and MC-MD to the ability of polarized water molecules to readjust during AT and have sufficient time to lose their transient dipoles.

An interesting application of the sampling methods of phospholipid mixtures would be an investigation of the asymmetric lipid bilayers. One can propose an AT that would exchange lipids between different layers. Since the flip/flop movements between the bilayers are an activated process with a significant barrier, such a move can greatly increase the equilibration rate. Of course, one should keep in mind that the membrane asymmetry is maintained in the biological systems by non-equilibrium processes, including ATP and gradient-powered transporters, and a true equilibrium may not be desired in such cases. However, for the investigation of synthetic systems, which may still be asymmetric, the MDAS algorithm and the Martini model are promising.

## VI. CONCLUSIONS

In the current paper, we explored the possibility of using the exchange-based MDAS and MC-MD algorithms for the efficient sampling of mixed lipid bilayers within the Martini and atomically detailed models. The model system is a binary 1:1 DPPC/DPPS mixture that illustrates how the advanced sampling approaches within the Martini force field can significantly increase the sampling and open new possibilities for simulations of large multi-component lipid mixtures. For the relatively small system considered in this paper (400 lipid molecules), the speedup factor can be as large as 11. In the case of the Martini model with polarizable water, the MC-MD approach yields low acceptance probabilities, making sampling with this approach inefficient. However, the MDAS method still provides up to 2.5 times speedup for the same setup. For exchange moves with small energy modifications, the use of the basic MC-MD sampling scheme might be a desirable approach. However, for more complex exchange moves that require substantial adjustments of the system, the MDAS algorithm is more efficient compared to the MC-MD approach. The importance of the proper design of an exchange move with the MDAS algorithm and the particular advantage of a coarser representation of lipid molecules in the Martini model is illustrated with the atomistic simulations of a DPPC/DPPS mixture, where the energy modification during an exchange move is significantly higher compared to the Martini model.

## Data Availability

The data that support the findings of this study are available from the corresponding author upon reasonable request.
